# Comparing the Safety and Efficacy of L-Glutamine, Voxelotor, and Crizanlizumab for Reducing the Frequency of Vaso-Occlusive Crisis in Sickle Cell Disease: A Systematic Review

**DOI:** 10.7759/cureus.24920

**Published:** 2022-05-11

**Authors:** Maurice H Dick, Arowa Abdelgadir, Vaishnavi Vijaya Kulkarni, Hamna Akram, Abanti Chatterjee, Sushil Pokhrel, Safeera Khan

**Affiliations:** 1 Medical School, Saint James School of Medicine, Arnos Vale, VCT; 2 Family Medicine, California Institute of Behavioral Neurosciences & Psychology, Fairfield, USA; 3 Internal Medicine, California Institute of Behavioral Neurosciences & Psychology, Fairfield, USA; 4 Neurology, California Institute of Behavioral Neurosciences & Psychology, Fairfield, USA; 5 Plastic Surgery, California Institute of Behavioral Neurosciences & Psychology, Fairfield, USA

**Keywords:** combination therapy, crizanlizumab, voxelotor, l-glutamine, vaso-occlusive crisis, sickle cell

## Abstract

Sickle cell disease (SCD) is a group of inherited red blood cell disorders affecting millions worldwide. The median life expectancy of someone with SCD remains significantly low despite improvements in standards of care and the implementation of hydroxyurea therapy. Notably, a 20-year interval existed (after the implementation of hydroxyurea therapy) prior to the approval of other sickle cell medications, namely, l-glutamine, voxelotor, and crizanlizumab. In this systematic review, these new medications' impact on the occurrences of vaso-occlusive crisis (VOC) events were analyzed and the adverse events of each were noted. Further, a secondary analysis was conducted to determine the effect of combination therapies, whether synergistic, antagonistic, or additive. The systematic review was conducted following the PRISMA 2020 guidelines. The effect-based and dose-effect-based approaches were utilized to determine the combined drugs combination index based on the recommended dosage to achieve an efficacy of 50%. L-glutamine and crizanlizumab were effective in reducing the frequency of VOC (p= 0.0216 and p = 0.02). Voxelotor effect on the reduction of VOC occurrences was not significant, however, its effect on increasing hemoglobin levels was significant (p= <0.001). In all three therapies, pain was the most common adverse event reported by participants. The analysis of combination therapies revealed that voxelotor plus l-glutamine was synergistic, voxelotor plus crizanlizumab was antagonistic, and l-glutamine plus crizanlizumab was additive. Thus, voxelotor plus l-glutamine combination therapy may be more beneficial to sickle cell disease patients. As such, robust combination drug studies for approved therapies used in SCD should be initiated with a specific focus on voxelotor plus l-glutamine. Additionally, the development of medications that lessen the pain burden in sickle cell disease patients should also be prioritized.

## Introduction and background

Sickle cell disease (SCD) is a group of inherited red blood cell disorders affecting millions worldwide and approximately 100,000 Americans [1]. SCD is associated with extensive multi-organ morbidity and an increased risk of early mortality [2]. The only treatment that can cure SCD is a hematopoietic stem cell transplant, which is only utilized in severe instances [3]. Hydroxyurea (HU) and improvements in standards of care within the past 10 years have led to an increase in the median life expectancy of persons with SCD from 28 years to 42-47 years [4, 5]. Nevertheless, the median life expectancy of persons with SCD remains significantly lower than the United States life expectancy of 78 years [6].

Almost one-third of SCD-related deaths occur suddenly and unexpectedly at home or within 24 hours of presentation to the hospital [7]. Adults with SCD are more likely to die from chronic complications, while children with SCD are more likely to die from acute complications [8]. The leading causes of death in SCD are vaso-occlusive crisis (VOC), cardiopulmonary disease, infection, and renal disease [4]. It is well established that hypoxemia exacerbates the manifestations of SCD. However, the consequences of chronic endothelial inflammation and repeated vaso-occlusive episodes have become more prevalent [4].

VOC is amongst the most common manifestations SCD patients present with to medical facilities [9]. VOC occurs when the polymerization of deoxygenated sickle hemoglobin (HbS) leads to the formation of sickle red blood cells (RBCs) [9]. Sickle RBCs coalesce (with other blood products), leading to small blood vessel occlusion, vascular inflammation, hypoxia, reperfusion injury, and ischemic tissue damage [9]. VOC is also associated with large-vessel intimal hyperplasia, thrombosis, and bone marrow fat embolization [9].

Hydroxyurea is a medication therapy that was approved by the US Food and Drug Administration (FDA) in 1998 for adults with SCD and later extended to patients two years of age and older in 2017. Voxelotor, l-glutamine, and crizanlizumab are more recently approved SCD pharmaceutical therapies that will be discussed further in this paper.

L-glutamine, approved in 2017, is indicated to reduce acute complications of SCD in patients five years old and older. The mechanism of action of l-glutamine in the treatment of SCD remains unknown; however, studies have shown it may lessen oxidative damage of sickled RBCs by improving nicotinamide adenine dinucleotide (NAD), a common oxidation-reduction (redox) cofactor, redox potential and increasing the availability of reduced glutathione [10]. The recommended dosage is 0.3 grams per kilogram of body weight per dose [10].

Voxelotor, approved in 2019 and indicated for treating SCD patients 12 years and older, interferes with the underlying pathology which modifies hematological parameters in SCD. It is an HbS polymerization inhibitor, and studies suggest it may inhibit RBC sickling, improve deformability, and reduce blood viscosity in SCD [11]. The recommended dosage of voxelotor is 1500 milligrams and it is administered orally once daily with or without food [11]. In SCD patients with severe hepatic disability, the recommended dose is 1000 mg once daily [11]. 

Crizanlizumab, approved in 2019, is indicated to reduce the frequency of VOC in SCD patients 16 years and older. It is a humanized IgG2 kappa monoclonal antibody that binds to P-selectin. P-selectin is an adhesion molecule implicated in inflammation, atherosclerosis, and coagulation and appears to be of particular importance in SCD [12, 13]. The recommended dose for crizanlizumab is 5 milligrams per kilogram and it is administered intravenously for 30 minutes on a week interval of zero, two, and every four weeks thereafter [14]. The recommended dosage form is an injection solution in a single dose ampoule of 100 milligrams per 10 milliliters [14].

To note, there was a 20-year gap prior to the development and approval of these relatively new SCD therapies. However, the FDA’s approval of l-glutamine, voxelotor, and crizanlizumab encouraged further therapeutic research, particularly in combination therapies for SCD [15]. Currently, over 30 treatment options exploring a diverse range of complementary approaches engineered for combination therapy are being studied in clinical trials to offer SCD patients multiple options and alternatives to the current quality-of-care medication hydroxyurea [15]. For example, the medicament LentiGlobin utilizes gene therapy and hematopoietic stem cell transplant. Functional human beta-globin genes are inserted ex vivo into the patient's hematopoietic stem cells then the modified cells are returned to the patient via stem cell transplantation. It is touted to be a game-changer for SCD patients who experience severe VOC events frequently (≥4 events in 24 months).

Despite the promising future of combination therapy, the development of treatment regimens has been slow and elusive [16]. A major challenge is designing and implementing an effective combination therapy regimen that is orally safe, accessible, and well-tolerated. To our knowledge, there is no published evidence evaluating combination therapies between voxelotor + l-glutamine, voxelotor + crizanlizumab, and l-glutamine + crizanlizumab, however, understanding the effectiveness and tolerability of these medicaments will further guide combination therapy and significantly influence the global burden of SCD.

This systematic review aims to explore the effect of voxelotor, l-glutamine, and crizanlizumab on SCD patients, specifically their response, efficacy, safety, and associated complications. A secondary analysis evaluating the possible safe, tolerable, and effective combinations between voxelotor, l-glutamine, and crizanlizumab based on their associated benefits and adverse events for the SCD patients was also conducted.

## Review

Methods

This systematic review was conducted following the Preferred Reporting Items for Systematic Review and Meta-Analyses (PRISMA) 2020 guidelines [17].

Data Collection and Search Strategy

A systematic search for articles and clinical articles published between 2017 and 2021 was conducted from Sept 20, 2021, to Sept 29, 2021, on PubMed, PubMed Central (PMC), MEDLINE, Cochrane, and ClinicalTrials.gov. A search strategy combining regular search terms and the Medical Subject Headings (MeSH) algorithm was applied. Regular keywords were employed for the search strategy for articles indexed in PubMed, PMC, and MEDLINE in the Cochrane Library and Clinicaltrials.gov. The databases, search strategies, and results are summarized in Table 1.

**Table 1:** Databases, Search Strategies and Search Results

Databases	Search Strategy	Search Results
PubMed, PMC, MEDLINE	Sickle Cell OR (((("Anemia, Sickle Cell/drug therapy"[Majr] OR "Anemia, Sickle Cell/prevention and control"[Majr])) AND ("Glutamine/administration and dosage"[Majr] OR "Glutamine/adverse effects"[Majr] OR "Glutamine/therapeutic use"[Majr] OR "Glutamine/toxicity"[Majr])) OR "voxelotor" [Supplementary Concept]) OR "crizanlizumab" [Supplementary Concept] OR Glutamine OR Voxelotor OR GBT440 OR crizanlizumab OR SelG1	73,238
Cochrane Library	Sickle cell AND glutamine OR voxelotor OR crizanlizumab	79
ClinicalTrials.gov	Sickle cell AND glutamine	6
	Sickle cell AND voxelotor	14
	Sickle cell AND GBT440	17
	Sickle cell AND crizanlizumab	9
	Sickle cell AND SelG1	1

Study Selection Eligibility

Two reviewers, MD and AA, independently identified and screened the collected articles for eligibility by title and then by abstract. Any conflict about eligibility was settled by the third reviewer, VK. The full text of relevant articles was then sought and assessed by the inclusion and exclusion criteria.

Inclusion and Exclusion Criteria

All studies (1) related to sickle cell disease, (2) from the past five years, 3) published in the English language, and 4) focusing on humans were included. All studies (1) not related to sickle cell disease, (2) published in other languages, (3) unpublished literature, (4) active clinical trials, (5) grey literature, (6) in-vitro studies, and (7) focusing on animals were excluded.

Bias and Quality Assessment

**Figure 1:**
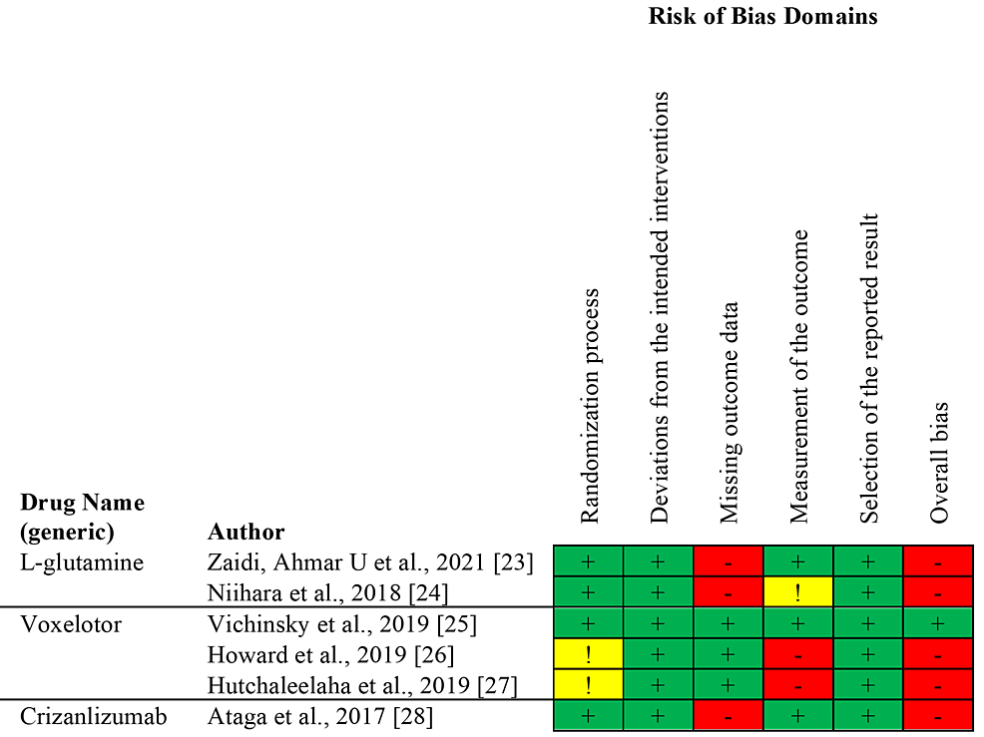
Cochrane Risk-of-Bias Tool for Randomized Trials (RoB 2). Risk of bias assessment: Low risk (+), Some Concerns (!), High Risk (-). This Figure contains information on the Cochrane Risk-of-Bias Tool for randomized trials to assess the quality and bias of the RCTs used in the systematic review.

**Figure 2:**
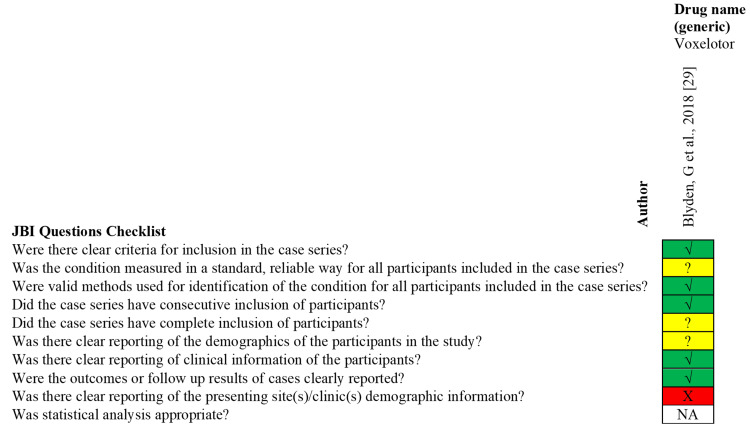
JBI Critical Appraisal Checklist for Case Series. Risk of bias assessment: Yes (√), No (X), Unclear (?), Not applicable (NA). This figure contains information on the JBI checklist for the case series included in the systematic review. The JBI checklist is a set of questions for case series to assess the quality of the studies.

**Figure 3:**
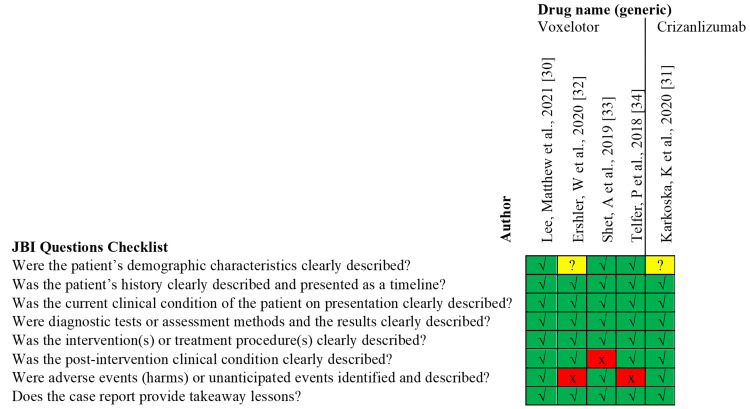
JBI Critical Appraisal Checklist for Case Reports. Risk of bias assessment: Yes (√), No (X), Unclear (?), Not applicable (NA). This figure is a summary of the JBI Critical Appraisal Checklist for Case Reports used to evaluate the quality and bias of the studies employed in the systematic review.

Eligible articles were independently evaluated for risk of bias by MD and HA. Conflicts were resolved by AC. Studies classified as high in bias in half or more of the assessment domains were excluded from this review. The tools used to assess the quality of the included studies are displayed in Table 2. Further, the Cochrane Risk of Bias Assessment Tool for the Randomized Control Trials (RCTs) is displayed in Figure 1, while the Joanna Briggs Institute (JBI) checklist for the case series and case reports are indicated in Figure 2 and Figure 3, respectively [18, 19].

**Table 2:** Quality assessment using the preferred checklists. (RCTs: Randomized Control Trials; JBI: Joanna Briggs Institute)

Type of Study	Tool Used	Number of Studies
RCTs	Cochrane Bias Assessment Tool [18]	6
Case Series	JBI Checklist for Case Series [19]	1
Case Reports	JBI Checklist for Case Reports [19]	5

Data Extraction

After screening the articles for bias and assessing their quality, data were extracted. The information extracted from the articles included the year of publication, study design, the purpose of the study, duration of the study, participation criteria, sample size, intervention, study outcomes, and treatment-related adverse events.

Selection of Combination Therapy

Combination therapies for SCD were suggested and analyzed based on the adverse events and benefits of the SCD therapies. The intent was to suggest combination therapies that display low toxicity, increased efficacy, and reduced chances of drug resistance development. Moreover, the toxicity threshold was evaluated for the combined drugs to propose safe SCD combination therapies. The suggested combination therapies were segregated based on benefits, adverse events, and drug administering requirements. Additionally, mathematical models were applied to obtain the combination index (CI) and determine the type of effect that would result from the combined drugs. The CI is a standard measure used to determine the degree of the drug interaction combination effect.

The effect-based and dose-effect-based approaches were utilized in this study to determine the interaction CI of the combined drugs based on the recommended dosage to achieve an efficacy of 50%. The effect-based approach compares the effect that results from combining the drugs against the individual effect of the therapies, while the dose-effect strategy considers the dosage of each drug concentration to the effect produced [20]. Similarly, the fractional product method will be applied to compute the combined effect of the drugs. The limitation of the fractional product method is that the effect cannot exceed 100% [21]. To apply the fractional product method in this study, the assumption made is that the SCD therapies have hyperbolic curves.

The Bliss Independence Model and Loewe Additivity Model were selected to calculate the CI of the combined SCD therapies. The Bliss Independence Model is an effect-based approach used in the analysis of combined drug data [20]. Loewe Additivity model is a dose-effect-based approach that was developed on the concept of the “sham mixture” of the drugs combined [20]. The sham mixture indicates that dose (b_a_) can be applied to any other dose (b) of medication B to give the combination an additive effect. The Bliss Independence Model is a regularly used substitute of Loewe Additivity; thus, providing the element of comparison of the findings.

Effect-Based Strategy via the Bliss Independence Model

The Bliss Independence Model utilizes the assumption that the drugs combined work independently and do not interfere with each other but produce the same effect. In the Bliss Independence Model, the probabilistic formula is given by E_a_ + E_b_ (1-E_a_) = E_a_ + Eb - E_a_E_b_, where 0 ≤ E_a_ ≤ 1 and 0 ≤ E_b_ ≤ 1 [20]. Ea is the effect of drug A, E_b_ is the effect of drug B, and E_a_E_b_ is the combined effect of the two drugs. The effect of the individual drugs is denoted by the percentage of VOC reduction and the CI formula is represented by CI = $\frac{E_{a}+E_{b}-E_{a}E_{b}}{E_{ab}}$ [20]. The limitation of using the CI to assess the combination of drug therapies in the Bliss Independence Model is that the probability values obtained only range from 0 to 1; 0 represents antagonism while 1 indicates additivity [20]. Antagonism means the combined drug therapy will have a lesser effect than the summed effect of the individual drugs while additivity means the combined drug will have a similar effect to the summed individual drugs effect.

Dose-Effect-Based Strategy via the Loewe Additivity

The Loewe Additivity Model applies the dose equivalence and sham combination principles. The dose equivalence principle states that for a particular effect, the dose (a) of drug A corresponds to dose (b_a_) of drug B and reciprocally. The sham combination principle states that (b_a_) can be added to any other dose (b) of drug B to produce the additive effect of the combination therapy [20]. However, in Loewe Additivity, the additive effect of the combined drugs is dependent on the individual dose-effect curve and is mathematically expressed as:

 Effect (a + b) = E_A_ (a + a_b_) = EB (b_a_ + b) = E_AB_, 

 E_A_ is derived from the dose-effect curve of drug A

 (a + a_b_) is analogous to dose A producing the effect (E_AB_) and respectively for drug B.

This assumes that the therapies have a constant potency ratio (R = $\frac{A}{B}$) with a constant ratio of doses at every level of effect. The relation among all pairs of doses (a, b) generating the aggregate effect E_AB_ and the single doses A and B essential to obtaining that effect can be subsequently defined; thus, on the basis of the dose-effect-based approach, the Loewe Additivity is given by $\frac{a}{A}+\frac{b}{B}$ = 1 [20]. The CI formula is represented by $\frac{a}{A}+\frac{b}{B}$; where CI < 1 means synergy, CI > 1 indicates antagonism and CI = 1 represents additivity [20]. Thus indicating, that the combination therapy would have a greater (CI < 1), lesser (CI > 1), or similar (CI = 1) effect than the expected additive effect. Chou (2011) also created a table, shown below in Table 3, which illustrates the description and symbols associated with a particular range of CI values [22].

**Table 3:** Description and Symbols of Synergism and Antagonism in Drug Combinations Analyzed Using CI Method.

Range of CI	Description	Graded Symbols
< 0.1	Very strong synergism	+ + + + +
0.1 – 0.3	Strong synergism	+ + + +
0.3- 0.7	Synergism	+ + +
0.7- 0.85	Moderate synergism	+ +
0.85- 0.90	Slight synergism	+
0.90- 1.10	Nearly additive	±
1.10- 1.20	Slight antagonism	-
1.20- 1.45	Moderate antagonism	- -
1.45- 3.3	Antagonism	- - -
3.3- 10	Strong antagonism	- - - -
>10	Very strong antagonism	- - - - -

Results

**Figure 4:**
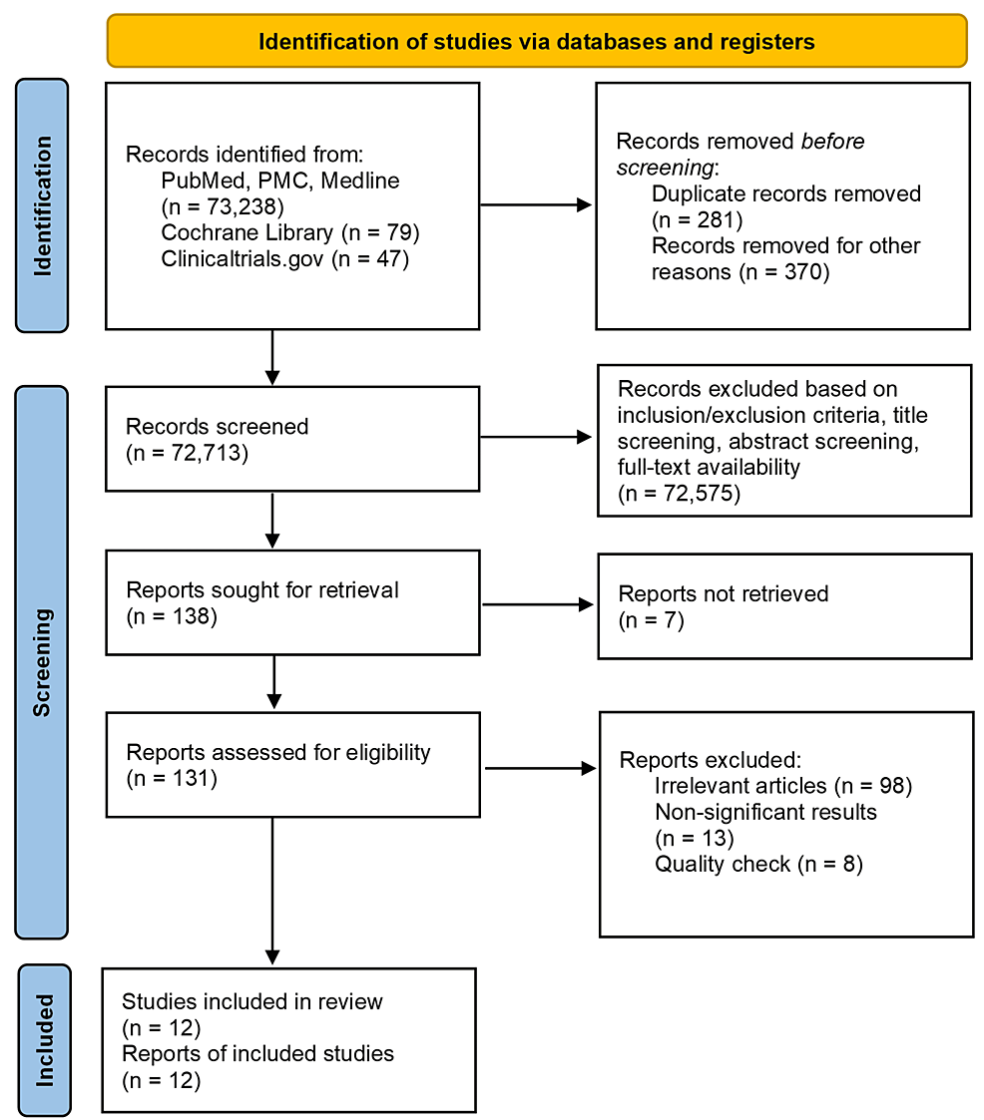
PRISMA flow diagram outlining the process used to identify and screen articles obtained from PubMed, PMC, Medline, Cochrane Library, and Clinicaltrials.gov and the subsequent selection of twelve articles included in the systematic review. From: Page MJ, McKenzie JE, Bossuyt PM, et al. The PRISMA 2020 statement: an updated guideline for reporting systematic reviews [17]. For more information, visit: http://www.prisma-statement.org/

A total of 73,364 articles were identified from the PubMed, Cochrane Library, and ClinicalTrials.gov; and 651 articles were removed mainly due to duplication. The number of records screened by automation was 72,713 and 72,575 were excluded. A total of 138 records were sought for retrieval, while seven of them were not retrieved; 131 records were screened for eligibility and 118 reports were excluded. Also, 12 articles were included in our systematic review after a detailed quality assessment was conducted to eliminate irrelevant articles. The search process in the form of a PRISMA flow diagram is depicted in Figure 4.

Study Characteristics

A total of 12 studies were reviewed. The reviewed articles employed various study designs such as RCTs (n=six), case series (n=one), and case reports (n=five). The articles employed either voxelotor, l-glutamine, or crizanlizumab as the intervention. All the RCTs contained a noteworthy percentage of participants who concomitantly continued their stable dosage of hydroxyurea treatment.

Randomized Clinical Trials

Study 1 [23] was a reanalysis of Study 5's [24] data, and the primary purpose was to determine the differences in the median annual rates of VOC between the treatment and placebo groups.

Study 2 was a Phase 3 RCT investigating voxelotor in 274 SCD patients aged 12 to 65 years. The inclusion criteria included SCD patients with a Hgb level of between 5.5g/dl to 10.5g/dl during screening and 1-10 VOC pain crises within the past 12 months. The study duration was 72 weeks, the interventions were Voxelotor 1500 mg (high-dose), 900 mg (low-dose), and placebo, and the primary purpose was to determine the percentage of participants with an increase in Hgb >1 g/dL from baseline to week 24. Patients receiving a stable dose of hydroxyurea were included [25].

Study 3 was a Phase one/two RCT investigating the safety and tolerability of voxelotor in 54 SCD patients aged 18 to 60 years with Hgb levels greater than 6.0 g/dL and less than 10.4 g/dL. Voxelotor 500 mg, 700 mg, 1000 mg were studied in the 28-day cohorts, daily voxelotor 700 mg and 900 mg were studied in the 90-day cohorts, and daily voxelotor 900 mg was studied for an additional 6 months in selected patients [26].

Study 4 was a Phase 1/2 RCT evaluating the safety, tolerability, pharmacokinetics, and pharmacodynamics of voxelotor in 40 healthy volunteers and eight SCD patients aged 18 to 55 years with Hgb levels greater than 6.0 g/dL and less than 10.4 g/dL. The study was partitioned into three designs: (1) single ascending dose (SAD) where healthy participants were started at 100 mg with subsequent dose escalations to 400, 1000, 2000, and 2800mg, and SCD patients were given a single dose of voxelotor 1000 mg or equivalent placebo; (2) multiple ascending dose (MAD) three cohorts of healthy volunteers received voxelotor doses of 300, 600 and 900, or matching placebo, for 15 days; (3) which included SCD patients only and the results were published in Howard et al. (2019) [27-28]. 

Study 5 was a Phase 3 RCT investigating l-glutamine in 230 SCD patients aged five to 58 years who had at least two documented pain crises during the previous year. The study duration was 48 weeks, and the primary purpose was to determine if l-glutamine 0.3 g/kg, two times daily by mouth, reduced the number of SCD crises. Patients receiving a stable dose of hydroxyurea were included and patients with renal and hepatic insufficiencies were excluded [24].

Study 6 was a Phase 2 RCT investigating crizanlizumab in 198 SCD patients aged 16 to 65 years who experienced two to 10 pain crises within the past 12 months. The primary purpose of the study was to determine the impact of crizanlizumab 2.5 mg/kg (low-dose), 5.0 mg/kg (high-dose) on the occurrence of VOC for a period of 52 weeks. Patients receiving a stable dose of hydroxyurea were included [28].

Case Series and Reports

Study 7 was a case series in which voxelotor 900 mg, or 1500 mg was given once daily for a duration of six to 17 months to seven SCD patients (three males and four females) aged 22 to 67 years with Hgb levels less than 6 g/dL, or with extreme or life-threatening health complications. All participants, according to the judgment of the treating physician, previously exhausted available treatment options i.e., hydroxyurea treatment. For each patient, investigators secured experimental new medication applications from the FDA, and treatment was in accordance with compassionate use voxelotor criteria [29].

Study 8 was a case report of a 53-year-old SCD female with a 5.0 g/dL Hgb level at baseline, pulmonary hypertension, and stage IV chronic kidney disease. She was on epoetin alfa and required red blood cell transfusions every three months to treat symptomatic anemia. Prior to her voxelotor 1500mg once daily treatment, her hydroxyurea treatment was put on hold due to reticulocytopenia [30].

Study 9 was a case study of a 17-year-old male with SCD who experienced frequent VOCs, adherence to hydroxyurea waned, and had five hospitalizations within the previous 12 months. Crizanlizumab 5 mg/kg was started to reduce the frequency and severity of pain episodes [31].

Study 10 was a case report of a 39-year-old SCD female patient diagnosed with COVID-19 and admitted to the hospital with complaints of skeletal pain unrelieved by oxycodone. On admission, her Hgb level dropped to 6.7 from 7.9 g/dL and there was no improvement in her Hgb level after infusion of two units of pRBCs. Subsequently, Voxelotor 1500 mg daily was initiated. The author did not mention if the patient was taking hydroxyurea [32].

Study 11 was a case report on a 38-year-old female with SCD and past medical history of ACS, chronic renal insufficiency, secondary iron overload, sickle hepatopathy, cardiomyopathy, cholelithiasis, osteomyelitis, and multi-organ failure. Her previous treatments, including hydroxyurea, decitabine, AES-103 (5-hydroxymethylfurfural, 5-HMF), omega-3 fatty acids, oral magnesium, pRBC transfusions, and erythropoietin, caused severe complications, did not improve her health nor show clinical benefits. Due to the failure of multiple treatments, voxelotor 1500mg was administered to the patient [33]. 

Study 12 was a case report involving a 27-year-old male SCD patient with jaundice that manifested as scleral icterus and required tinted glasses. He stopped hydroxyurea treatment 12 months prior to the study and participated in a Phase 2 clinical trial's (NCT03041909) follow-on extensions where he received voxelotor 900 mg daily for six months [34].

Main Findings

Herein, we review 12 studies that investigated the impact of voxelotor, l-glutamine, and crizanlizumab on SCD-related complications in 347, 230, and 199 SCD patients, respectively. Additionally, one study focusing on the safety of voxelotor included 40 healthy volunteers. The studies employed various statistical methods which validated that l-glutamine and crizanlizumab were effective in the management of VOC and voxelotor was effective in improving hemoglobin levels.

Randomized Clinical Trials

The findings of the RCTs utilized in this systematic review are presented in Table 4 and highlighted in the subsections below.

**Table 4:** Evidence Table of the RCTs Reviewed ACS- acute chest syndrome, HbSβ0- sickle beta zero thalassemia, HbSβ+- sickle beta plus thalassemia, HbSC- sickle hemoglobin C disease, HbSS- homozygous hemoglobin S, Hgb- hemoglobin, RCT- randomized controlled trial, SCD- sickle cell disease, VOC- vaso-occlusive crisis.

Study	Author (year)	Study Design (phase)	Primary Purpose	Duration of Study	Participants’ criteria	Sample Size	Intervention	Outcome of the Intervention	P-value
1	Zaidi et al. (2021) [23]	Reanalysis of RCT (Phase 3)	To determine the differences in the median annual rates of VOC between the treatment and placebo	48 weeks	Sickle cell anemia (homozygous hemoglobin S [HbSS]), or sickle beta zero thalassemia (HbSβ^0 ^).	230	l-glutamine 0.3g/kg, two times daily by mouth	Reduced the rate of VOCs	p= 0.0216
								Reduced the number of days hospitalized for VOC	p= 0.042
								Reduced rates of ACS	p= 0.003
2	Vichinsky et al. (2019) [25]	Randomized Controlled Trial (Phase 3)	To determine the percent of participants with increase in Hgb >1 g/dL from baseline to week 24 (in the intention-to-treat analysis)	72 weeks	Sickle cell disease (homozygous hemoglobin S, sickle hemoglobin C disease, hemoglobin Sβ thalassemia, or other genotypic variants of SCD).	274	Voxelotor low-dose (900 mg), high-dose (1500 mg)	high dose voxelotor group Hgb increased >1.0 g/dL	p <0.001
								high dose voxelotor group mean Hgb increased by 1.1 g/dL	p <0.001
								indirect bilirubin level was reduced in the high dose voxelotor group	p < 0.001
								percentage of reticulocytes was reduced in the high dose voxelotor group	p < 0.001
								adverse events	reported
3	Howard et al. (2019) [26]	Randomized Controlled Trial (Phase 1/2)	Evaluating the safety, tolerability, pharmacokinetics, and pharmacodynamics of voxelotor and frequency of VOC in SCD patients.	Varies	HbSS genotype or HbSβ^0^	54	Voxelotor (GBT 440) 28 days (500, 700, or 1000 mg), 90 days (700 or 900 mg)	voxelotor increased Hgb levels compared to placebo	p <0.05
								voxelotor decreased sickled red cells compared to placebo	p <0.05
								voxelotor decreased reticulocyte counts compared to placebo	p <0.05
								voxelotor decreased unconjugated bilirubin levels decreased compared to placebo	p <0.05
								adverse events	reported
4	Hutchaleelaha et al. (2019) [27]	Randomized Controlled Trial (Phase 1/2)	Evaluating the safety, tolerability, pharmacokinetics, and pharmacodynamics of voxelotor	15 days	Healthy volunteers (n= 40) SCD (HbSS, HbSβ^0^, HbSβ^+^ or HbSC) patients (n= 8).	48	Voxelotor (GBT 440) Part A: single dose in healthy volunteers (2800 mg) and SCD (1000mg); Part B: multiple doses (up to 900 mg) in healthy volunteers	single doses of voxelotor were well tolerated up to 2800 mg in healthy volunteers (maximum undetermined)	not reported
								Voxelotor increased Hgb oxygen affinity	not reported
								voxelotor did not impair oxygen delivery	not reported
								adverse events	reported
5	Niihara et al. (2018) [24]	Randomized Controlled Trial (Phase 3)	To determine if l-glutamine reduces the number of SCD crises	48 weeks	Sickle cell anemia (homozygous hemoglobin S [HbSS]), or sickle beta zero thalassemia (HbSβ^0 ^).	230	l-glutamine 0.3g/kg, two times daily by mouth	Decreased the frequency of SCD crises	p = 0.005
								Decreased the frequency of hospitalizations	p = 0.005
								Decreased the occurrences of acute chest syndrome	p = 0.003
								Decreased the length of stay in hospital	p = 0.02
								Prolonged the time to the first crisis	p = 0.02
								Prolonged the time to the second crisis	p = 0.03
								adverse events	reported
6	Ataga et al. (2017) [28]	Randomized Controlled Trial (Phase 2)	To determine the impact of crizanlizumab on occurrence of VOC.	52 weeks	Sickle cell disease (homozygous hemoglobin S [HbSS], sickle hemoglobin C disease [HbSC], sickle beta zero thalassemia [HbSβ^0^], sickle beta plus thalassemia [HbSβ^+^] or other genotypes); between 2-10 VOC in the past 12 months.	198	Crizanlizumab low-dose (IV 2.5mg/kg), high-dose (IV 5.0mg/kg)	high dose crizanlizumab decreased the frequency of VOC	p = 0.02
								high dose crizanlizumab prolonged the time to the first crisis	p = 0.001
								high dose crizanlizumab prolonged the time to the second crisis	p = 0.02
								High dose crizanlizumab reduced the frequency of uncomplicated crises	p = 0.02
								adverse events	reported

L-glutamine and crizanlizumab reduced VOC episodes: l-glutamine and crizanlizumab were effective in prolonging the time between the first and second pain crisis. Additionally, both decreased the frequency of VOC among SCD patients. Voxelotor had no significant effect on VOC.

In Study 1, there was a 45% lower median annual rate of VOC in the l-glutamine group compared to the placebo group (2.37 vs 4.30, p = 0.0216) [23]. In Study 5, the median rate of VOC in the l-glutamine treatment group was significantly reduced in comparison to the placebo (3.0 vs 4.0, p = 0.005). L-glutamine considerably prolonged the median time of the first pain crisis (84 vs. 54 days, p = 0.02) and second pain crisis (212 vs 133 days, p = 0.03) [24].

In Study 6, the annual median rate of VOCs was 1.63 for the high dose crizanlizumab group against placebo at 2.98, representing a 45.3% lower rate (p = 0.01). The median time to the first crisis (4.07 months, p = 0.001) and second crisis (10.32 months, p = 0.02) was significantly prolonged compared to the placebo (1.38 and 5.09, respectively). Additionally, high dose crizanlizumab was associated with lower annual rates of uncomplicated pain crises (1.08 vs 2.91), representing a 62.9% lower rate (p = 0.02) [28].

Study 2 analysis of voxelotor's effect on the annual VOC incidence rate did not yield any significant results. Among participants who had at least two crises in the previous year, the incidence rate was 2.88, 3.39, and 3.50 in the 1500 mg voxelotor, 900 mg voxelotor, and placebo groups, respectively. This represented a 0.62 difference and 17.7% rate reduction in the 1500 mg voxelotor group compared to the placebo group. The incident rate among all participants in the study was 2.77, 2.76, and 3.19 in the 1500 mg voxelotor, 900 mg voxelotor, and placebo groups, respectively [25].

L-glutamine reduced the occurrence of hospitalizations and the number of days hospitalized per year: in Study 1, the median annual number of days hospitalized was reduced (8.45, p = 0.042) in patients prescribed l-glutamine compared to the placebo group (13.17). This represented a 4.72-day difference and a 36% reduction [23]. In Study 5, there were fewer hospitalizations (median rate of 2.0 vs 3.0, p = 0.005) and fewer hospital days (cumulative median 6.5 vs 11, p = 0.02) in the l-glutamine therapy group compared to placebo [24].

L-glutamine significantly decreased the occurrences of ACS in SCD: based on the data in Study 1, the annual rates of ACS decreased by 55% in favor of l-glutamine therapy (0.18, p = 0.003) compared to placebo at 0.40 [23]. In Study 5 there was a 14.5% reduction (p = 0.003) in ACS incidences in the l-glutamine group compared to placebo [24].

Voxelotor increased hemoglobin levels and decreased sRBC and reticulocytes levels: In Study 2, the high dose voxelotor group had an increase in Hgb greater than 1.0 g/dL (p <0.001) and a greater mean hemoglobin increase compared to placebo (1.1 vs -0.1 g/dL, p <0.001). Additionally, there was a 24.4% difference (p <0.001) in the reduction of reticulocytes between the high-dose voxelotor and placebo groups [25].

In Study 3, voxelotor increased hemoglobin levels (1.0 vs -0.1, p <0.05), and decreased reticulocytes (-12.9 vs 0.5, p <0.05), and sickled red cells (-74.0 vs 6.9, p <0.05) compared to the placebo group [26]. In Study 4, a single dose of voxelotor up to 2800 mg was well tolerated in healthy patients; the maximum was not determined. Voxelotor increased oxygen affinity without compromising oxygen delivery [27].

Voxelotor decreased unconjugated bilirubin levels: in Study 2, there was a 25.9% difference (p <0.001) in the reduction of indirect bilirubin levels between the high dose voxelotor group and placebo [25]. In Study 3, voxelotor decreased unconjugated bilirubin levels compared to placebo (-39.7 vs 14.8, p <0.05) [26].

Safety of l-glutamine treatment: in Study 5, adverse events with the greatest incidence include noncardiac chest pain, back pain, pain in limbs, fatigue, and nausea. Other treatment-related adverse events include headache, dizziness, tachycardia, upper abdominal pain, urinary tract infection (UTI), nasal congestion, vomiting, diarrhea, and constipation. Two patients with complicated past medical histories died while receiving l-glutamine. Seven participants receiving l-glutamine therapy withdrew due to (dyspepsia; hot flashes; burning sensation in the feet; hypersplenism and abdominal pain) adverse events (four participants), or pregnancy (three participants) [24].

Safety of voxelotor treatment: in Study 2, the incidence of adverse events was greater in the high and low-dose treatment groups compared to placebo (94% and 93% vs 89%, respectively). Among the adverse events reported, headache and diarrhea were the most common. Other adverse events reported were arthralgia, upper respiratory tract infection, abdominal pain, fatigue, rash (generalized, erythematous, urticaria, papular, macular, maculopapular, vesicular, and pruritic), pyrexia, pain in extremity, back pain, vomiting, pain, noncardiac chest pain, upper abdominal pain, nausea. Four deaths, unrelated to the trial (according to the authors), occurred during the study [25].

In Study 3, the most reported adverse events were rash, headache, and diarrhea. Whilst drug rashes occurred in two patients, the other incidences of rashes were not considered to be drug rashes. Other adverse events include cough, general pain, back pain, and pain in the extremities. Five patients required dose reductions due to (1) grade 2 papular pruritic rash, (2) elevated liver enzymes, (3) abdominal discomfort, (4) nausea, and (5) an increase in Hgb >2 g/dL from baseline. One patient discontinued treatment due to a Grade 2 rash and no deaths were reported by the authors [26]. 

In Part A of Study 4, rash, headache, arthralgia, upper respiratory tract infection, and diarrhea were the adverse events reported by healthy individuals, whilst pain, nausea, vomiting, and diarrhea were reported by SCD patients. In Part B, headache, dizziness, abdominal pain, gastroenteritis, and diarrhea were the adverse events reported. One patient discontinued the study due to rash and another due to abdominal pain, diarrhea, and headache requiring analgesic treatment. No deaths were reported by the authors [27].

Saftey of crizanlizumab treatment: in Study 6, 27.8% of participants recorded serious adverse events (38 patients in the intervention group and 17 in the placebo). Pyrexia and influenza were common serious adverse events in treatment groups (high dose and low dose, respectively) and occurred at higher rates compared to placebo. Pneumonia was a serious adverse event reported in all groups. Other treatment-related adverse events were chest, back, limb, and musculoskeletal pain, headache, arthralgia, upper respiratory and urinary tract infections, pruritus, nausea, vomiting, and diarrhea. Five patients died during the trial, three in the treatment groups and two in the placebo. In the low dose treatment group, two single adverse events, anemia and intracranial hemorrhage (attributed to ketorolac) occurred whilst sepsis occurred in the placebo group [28].

Case Series and Reports

The findings of the case series and case reports interpreted in this study are displayed in Table 5 and accentuated in the subsections below.

**Table 5:** Evidence Table of the Case Series and Case Reports Reviewed AKI- acute kidney injury, CKD- chronic kidney disease, COVID- coronavirus disease, DRESS- drug rash with eosinophilia and systemic symptoms, EPO- erythropoietin, HbSβ^0^- sickle beta zero thalassemia, HbSS- homozygous hemoglobin S, Hgb- hemoglobin, HU- hydroxyurea, RBC- red blood cell, RCT- randomized controlled trial, SaO2- arterial oxygen saturation, SCD- sickle cell disease, sC5b-9- soluble Complement 5b-9, VOC- vaso-occlusive crisis.

Study	Author (year)	Study Design	Primary Purpose	Duration of Study	Participants’ criteria	Sample Size	Intervention	Outcome
7	Blyden et al. (2018) [29]	Case Series	compassionate use activity	6 to 17 months	HbSS (6), HbSβ^0^ (1) with <6.0 g/dL or life-threatening complications.	7 (male- 3, female- 4)	voxelotor 900 mg/day, 1500 mg/day	Improved Hgb levels by a mean of 1.8 g/dL at 24 weeks
								Decreased transfusions by 60%
								Decreased the number of VOC hospitalizations by 67%.
								Improved SaO2 from <95% to 98-99% (allowing discontinuation of long-term oxygen treatment).
								Improved distance walked, post-walk pulse rate and post-walk room air oxygen partial pressure on a 6-minute walk test by +57 meters, -7bpm, and +10% respectively at week 24.
								Improved depression
								One report of grade 2 diarrhea with 1500 mg (resolved upon return to 900mg)
								One report of grade 1 diarrhea with 900 mg which resolved with no treatment change.
								No serious adverse events were reported.
8	Lee et al. (2021) [30]	Case Study	started voxelotor when HU was put on hold due to reticulocytopenia	36 days, held for 14 days, then took for 40 additional days	HbSS, African American woman, pulmonary hypertension, and CKD Stage IV	1	voxelotor 1500 mg orally daily (36d), 1000 mg orally daily	Caused AKI with creatine of 5.32 mg/dL (up from 2.5 mg/dL baseline) and eosinophilia (15% on CBC w/ diff) on day 36 of the 1500mg dosing regimen.
								Caused DRESS (drug reaction with eosinophilia and systemic symptoms) on day 40 of the 1000mg dosing regimen.
9	Karkoska et al. (2020) [31]	Case Study	To describe a severe crizanlizumab infusion-related adverse effect	8 days	HbSS; adherence to HU waned.	1	crizanlizumab 300 mg (5mg/kg) infusion over 30 minutes IV (peripheral)	Initiation dose caused sudden and severe pain (10/10) in the back, legs, and head within 10 minutes. Infusion was stopped with approximately 30% of the drug administered.
								Desaturated (94% to 88%), and anemia worsened (7.3 g/dL to 6.7 g/dL) required oxygen supplementation and pRBCs transfusion, respectively.
								Elevated sC5b-9 levels
								Hemolysis, hypertransaminasemia, and renal phosphorous wasting was reported.
								Persistent fever to ≥39°C through day 5 without identifiable source was reported.
								Patient-reported the event as the most severe pain of his life and discontinued further crizanlizumab treatment.
								The patient reported stress due to the pain crisis event.
10	Ershler et al. (2020) [32]	Case Study	To spare RBC units during a COVID pandemic	15 days (5/4 to 5/19)	HbSS, COVID pneumonia, female.	1	voxelotor 1500 mg orally daily	Hgb improved from 6.5 to 10.4 g/dL.
								Avoided the need for additional RBC transfusions.
								Avoided the need for exchange transfusion.
11	Shet et al. (2019) [33]	Case Study	Compassionate basis therapeutic trial	6 months	HbSS, female, Nigerian	1	voxelotor 1500 mg/day	Had no change in labs nor clinical course; neither with spit dosing nor dose escalation to 1800 mg/day (transient elevation in serum EPO noted). Tapered and discontinued.
								Adverse effects reported were headache and grade 2 diarrhea.
12	Telfer et al. (2018) [34]	Case Study	Follow-on trial (structured 60-minute in-depth interview)	6 months	SCD, NCT03041909 RCT trial participant, male, black.	1	Voxelotor 900 mg by mouth once daily	Hgb improved from 9.9 g/dL to 11.1 g/dL
								Total bilirubin was reduced
								Unconjugated bilirubin reduced by 76%
								The patient reported: stoppage of minor crises relief of pain clearing of conjunctival icterus (within one week) reduction in stress no adverse events

Voxelotor reduced the occurrence and days of hospitalization for VOC pain: in Study 7, there was a 67% reduction in the number of hospitalizations for VOC pain-related cases, and the days of hospitalization were reduced by half for four patients. Additionally, two patients had no record of hospitalization within 17 months of the treatment [29].

Voxelotor reduced the rate of blood transfusion: in Study 7 [29], the rate of blood transfusion was reduced by 60%.

Voxelotor increased Hgb oxygen saturation: in Study 7 [29], four patients with a baseline SaO2 <95%, improved to 98-99%, resulting in two discontinuing long-term oxygen treatment. A patient with a 330 meters distance walked, 102 beats per minute post-walk pulse rate, and 86% post-walk room air oxygen partial pressure baseline on a six-minute walk test improved to 387 meters, 93bpm, and 96% at 24 weeks respectively.

Voxelotor reduced SCD depression: in Study 7, via the Patient Health Questionnaire-9, one patient with moderate depression improved to no/minimal depression, and another patient with moderately severe depression improved to mild depression [29]. 

Voxelotor increased hemoglobin levels: in Study 7, there was a mean increase in hemoglobin levels by 1.8 g/dL at week 24 weeks with hemoglobin levels increasing as early as two days for some patients. In Study 10 [32], the hemoglobin level increased from 6.5 g/dL to 8.0 g/dL in two days and to 10.4 g/dL on Day 15. In Study 12 [34], hemoglobin increased by 1.2 g/dL (from 9.9 to 11.1g/dL) in 90 days [29]. 

Voxelotor circumvented the need for additional RBC transfusions and or need for exchange transfusions: in Study 10 [32], the need for additional RBC transfusions and or exchange transfusions was circumvented.

Voxelotor had no effect on the patient in Study 11: The patient had no change in labs nor clinical course; neither with spit dosing nor dose escalation to 1800 mg/day (transient elevation in serum EPO noted). Voxelotor was tapered and discontinued [33].

Patient-reported, voxelotor decreased VOC Pain: in Study 12, the patient reported a stoppage in the occurrences of pain crisis and relief of SCD-related pain [34].

Voxelotor reduced unconjugated and conjugated bilirubin levels: in Study 12, there was a reduction in conjugated bilirubin and a decrease in unconjugated bilirubin by 76% [34].

Patient-reported, voxelotor cleared conjunctival icterus: in Study 12, the patient reported clearing of conjunctival icterus within one week and subsequently relinquished the need for tinted glasses [34].

Voxelotor decreased SCD-related stress of the patient in Study 12: the patient reported to be happier, had a reduction in stress, and worried less about pain crisis [34].

Safety of voxelotor: With regards to voxelotor, in Study 7 the adverse events recorded were grade 1 diarrhea in the 900mg treatment group and grade 2 diarrhea in the 1500mg cluster. The grade 1 diarrhea resolved without a change in the treatment plan and grade 2 diarrhea required a dose reduction. Two patients with preexisting end-stage organ damage died during the study [29].

In Study 8, after four weeks of voxelotor 1500mg once daily, the patient experienced acute kidney injury (AKI) with creatine of 5.32mg/dl and eosinophilia with 15% on CBC differential and an absolute count of 1.1 thousands/mm3. The treatment was stopped and resumed after two weeks when the AKI resolved, and the eosinophilia reduced. The voxelotor treatment dosage was reduced to 1000mg. After five weeks of treatment, the patient experienced facial swelling, cough, fatigue, fever, and pruritic rash around her lower extremities, back, chest, shoulders, neck, and face. Additionally, the patient laboratory test indicated a 2.5g/dL drop in hemoglobin levels, elevated WBC count at 13,900 mm3, eosinophilia at 5200 mm3, acute kidney injury with a creatinine of 3.96mg/dl, elevated bilirubin at 2.0 mg/dL and elevated liver enzymes (alkaline phosphatase-243, aspartate aminotransferase-129, alanine aminotransferase-82). The patient was diagnosed with voxelotor induced drug reaction with eosinophilia and systemic symptoms (DRESS) [30].

In Study 10, no adverse effects were reported [32]. In Study 11, the patient experienced headaches and diarrhea [33]. In Study 12, the patient reported the return of chronic pain and jaundice after the discontinuation of voxelotor therapy [34].

Safety of crizanlizumab: In Study 9 [31], the patient experienced severe pain in the head, back, and legs within 10 minutes of the initial crizanlizumab therapy. Treatment was subsequently stopped with approximately 30% of the appropriate dose administered. The pain continued to worsen, and the patient required hospital admission after developing a persistent fever of 39.3 °C (102.7 °F) and a drop in oxygen saturation to 88%. The patient was diagnosed with acute chest syndrome. Throughout the seven-day hospital stay, the patient’s pain and fever proved difficult to manage and laboratory testing revealed an elevated soluble Complement 5b-9 (sC5b-9) level of 258 ng/mL, and a drop in hemoglobin from 7.8 to 6.7 g/dL and reticulocyte count from 304 to 179 x109/L. Furthermore, hypophosphatemia (3.2 mg/dL on admission to 1.3) was noted, in addition to significant elevations in nucleated RBC from 0.96 to 2.26 x103/mcL, total bilirubin from 3.7 to 4.5 mg/dL, alkaline phosphatase from 111 to 349 unit/L and ALT from 39 to 150 unit/L. AST levels only increased by two (from 77 to 79 unit/L). After discharge, the patient reported continued pain and analgesic use, as well as significant psychological stress with nightmares of the event [31].

Selection and Evaluation of Combination Therapies

**Figure 5:**
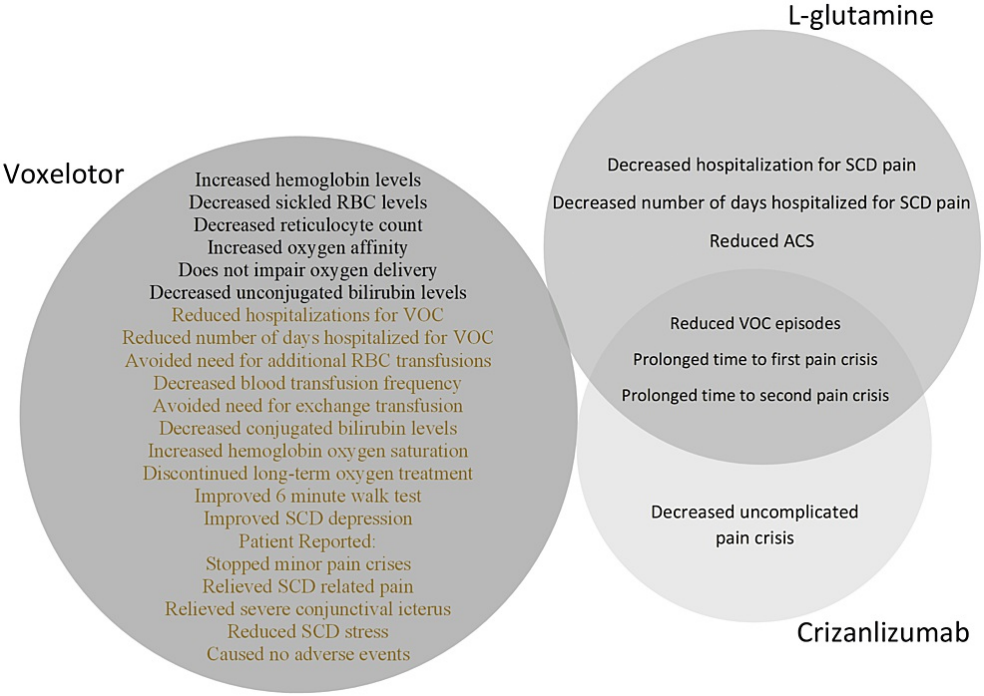
A Venn Diagram of the benefits of the SCD therapies from the articles used in the systematic review. Similar effects between the drugs are included. The adverse events reported in the randomized controlled trials are denoted in black text whilst the adverse events reported in the case series and case reports are denoted in colored text. ACS- acute chest syndrome, RBC- red blood cell, SCD- sickle cell disease, VOC- vaso-occlusive crisis.

**Figure 6:**
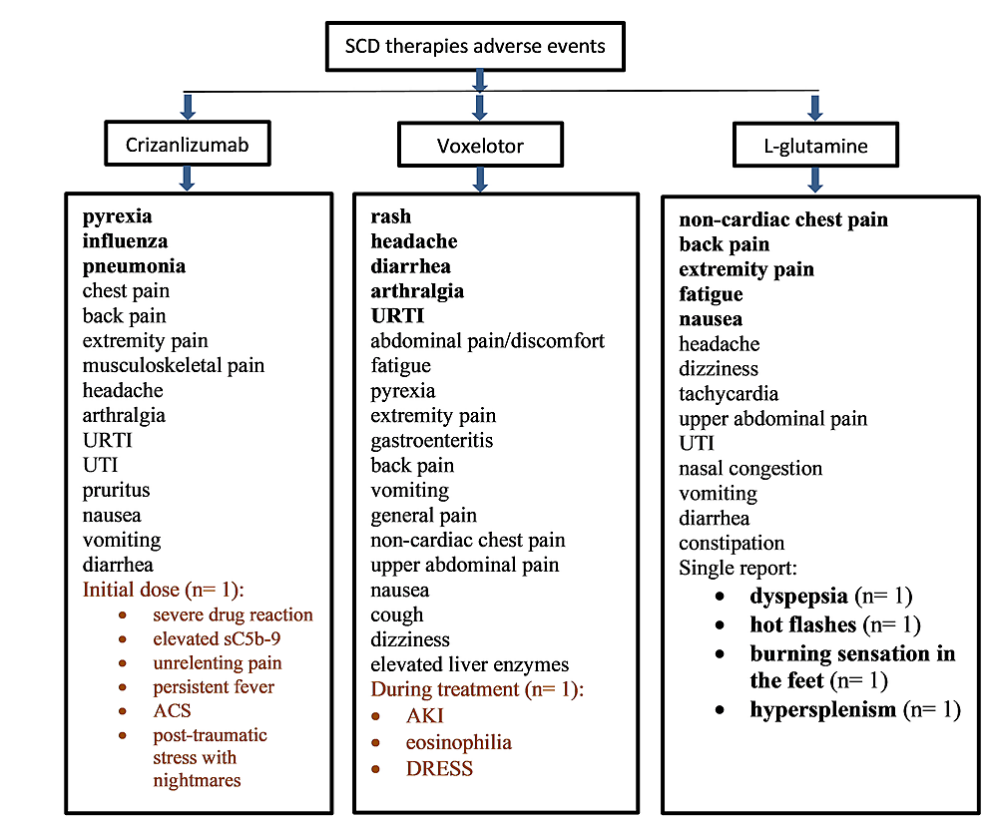
A summary of voxelotor, crizanlizumab, and l-glutamine adverse effects from the 12 eligible articles used in the systematic review. The adverse events reported in the randomized controlled trials are denoted in black text whilst the adverse events reported in the case series and case reports are denoted in colored text. Major adverse events are denoted in bold text. ACS- Acute Chest Syndrome, AKI- acute kidney injury, DRESS- drug reaction with eosinophilia and systemic symptoms, n- the number of cases, rash- (generalized, erythematous, urticaria, papular, macular, maculopapular, vesicular, pruritic), sC5b-9- soluble Complement 5b-9, URTI- upper respiratory tract infection, UTI- urinary tract infection.

**Figure 7:**
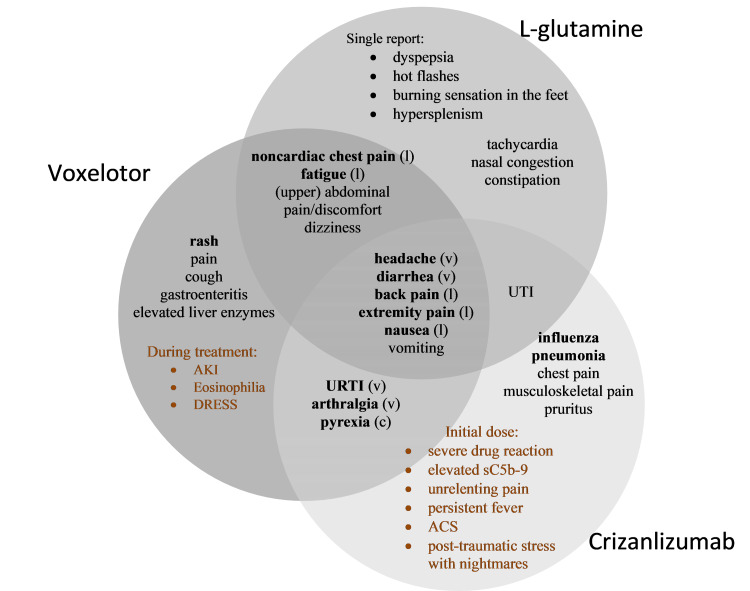
A Venn Diagram of the adverse events (of named drugs) reported in the 12 eligible articles used in the systematic review. The adverse events reported in the randomized controlled trials are denoted in black text whilst the adverse events reported in the case series and case reports are denoted in colored text. Major adverse events are denoted in bold text. ACS- Acute Chest Syndrome, AKI- acute kidney injury, c- major adverse event associated with crizanlizumab, DRESS- drug reaction with eosinophilia and systemic symptoms, l- major adverse event associated with l-glutamine, rash- (generalized, erythematous, urticaria, papular, macular, maculopapular, vesicular, pruritic), sC5b-9- soluble Complement 5b-9, URTI- upper respiratory tract infection, UTI- urinary tract infection, v- major adverse event associated with voxelotor.

Among the three therapies, voxelotor had the greatest number of unique and statistically significant positive outcomes (See Figure 5), however, the adverse effects profile of all three therapies were notable and somewhat tolerable (See Figures 6-7).

Voxelotor RCT Study 2 [25], l-glutamine RCTs Study 5 [25] and Study 6 [23], and crizanlizumab RCT Study 6 [28] were selected to determine the efficacy of possible combination therapies to reduce VOC pain in SCD patients. For l-glutamine, Study 5 [25] was selected because it was the original study, while Study 1 [23] had the annual VOC rates required for the CI calculation.

Prior to conducting the Bliss Independence Model and Loewe additivity, the units of the drugs were converted to a standard measure to help in determining the dose ratio for the therapies. For voxelotor, the recommended dose is 1500 mg for adults and pediatric patients 12 years and older who weigh 40 kg or greater; thus, 1500 mg/ 40 kg is equivalent to 37.5 mg/kg. For L-glutamine, the unit used in the studies was g/kg, thus 0.3g/kg is equivalent to 300mg/kg. In the crizanlizumab study, the dose was expressed in mg/kg; thus, no conversion was required.

Bliss Independence Model

The recommended dosage for voxelotor is 1500mg (37.5 mg/kg), while l-glutamine is 0.3 g/kg (300 mg/kg) and crizanlizumab is 5 mg/kg. Vichinsky et al. (2019), Zaidi et al. (2021) and Ataga et al. (2017) were selected to represent VOC pain reduction in percentage form, 17.7% (voxelotor), and 45% (l-glutamine), and 45.3% (crizanlizumab), respectively [23, 25, 28]. Combination Index (CI) is the standard measure of combination drug effect that designates a greater (CI < 1), lesser (CI > 1) or similar (CI = 1) effect than the expected additive effect.

Voxelotor + Crizanlizumab

The voxelotor drug effect will be represented by E_a_ and crizanlizumab by E_b_.

E_50, a_ + E_50, b_ (1-E_a_) = E_50, a_ + E_50, b_ - E_a_E_b_,

E_a_= 17.7% (0.177), E_b_ = 45.3% (0.453).

CI = $\frac{E_{a}+E_{b}-E_{a}E_{b}}{E_{ab}}$.

The additive effect was computed using the fractional product method [21].

(1 - 0.177) × (1- 0.453) = 0.450

(1 - 0.450) = 0.55

CI = $\frac{0.177+0.453-(0.177\times 0.453)}{0.55}$ = 1

The CI obtained for voxelotor + crizanlizumab is 1; hence, this combination treatment has an additive effect in the reduction of VOC pain crisis among SCD patients.

Voxelotor + L-glutamine

The voxelotor drug effect will be represented by E_a_ and l-glutamine by E_b_.

E_50, a_ + E_50, b_ (1-E_a_) = E_50, a_ + E_50, b_ - E_a_E_b_,

E_a_= 17.7% (0.177), E_b_ = 45% (0.45).

CI = $\frac{E_{a}+E_{b}-E_{a}E_{b}}{E_{ab}}$.

The additive effect was computed using the fractional product method [21].

(1 - 0.177) × (1- 0.45) = 0.45

(1 - 0.45) = 0.55

CI = $\frac{0.177+0.45-(0.177\times 0.45)}{0.55}$ = 1

The CI = 1, the combination of voxelotor and l-glutamine results in an additive effect.

Crizanlizumab + L-glutamine

The crizanlizumab drug effect will be represented by E_a_ and l-glutamine by E_b_.

E_50, a_ + E_50, b_ (1-E_a_) = E_50, a_ + E_50, b_ - E_a_E_b_,

E_a_= 45.3% (0.453), E_b_ = 45% (0.45).

CI = $\frac{E_{a}+E_{b}-E_{a}E_{b}}{E_{ab}}$.

The additive effect was computed using the fractional product method [21].

(1 - 0.453) × (1- 0.45) = 0.30

(1 - 0.30) = 0.7

CI = $\frac{0.453+0.45-(0.453\times 0.45)}{0.7}$ = 1

The combination therapy between the crizanlizumab and l-glutamine has an additive effect.

Loewe Additivity Model

Voxelotor + Crizanlizumab

Dose a (d_a_) will be represented by voxelotor 37.5 mg/kg (1500mg), while dose b (d_b_) is crizanlizumab 5.0 mg/kg. Drug A is voxelotor and has a 17.7% effect on VOC pain crisis reduction and B is crizanlizumab with 45.3%.

The constant potency ratio (R) for drug A relative to drug B can be calculated by R = $\frac{A}{B}$, where A and B are the effect sizes of the SCD therapies; hence A = 17.7% and B = 45.3%. The potency can be calculated by assessing the effect (f) of the drugs combined, D_ea_ representing the effect of drug A which is 17.7% and D_eb_ is 45.3%; thus, the potency is R = $\frac{0.177}{0.453}$ = 0.391. 

Loewe additivity of drug A (D_ea_= 17.7%) with response to drug B (D_eb_= 45.3%) is

d_a_ + d_a_D_ea_/D_eb_ or d_a_/D_eb_ + d_b_/D_eb_ = 1

d_a_ + d_b_D_ea_/D_eb_ = 37.5 + ($\frac{5\times 0.177}{0.453}$) = 39.454= A

d_a_/D_ea_ + d_b_/D_eb_ = $\frac{37.5}{0.177}+\frac{5}{0.453}$ = 222.9= B

 CI = $\frac{a}{A}+\frac{b}{B}$ = $\frac{37.5}{39.454}+\frac{5}{222.9}$ = 0.9

The CI obtained is less than 1; hence, synergism. However, based on Table 3, CI = 0.9 is described as nearly additive. The combination of voxelotor and crizanlizumab may cause an additive effect or improve the effectiveness of the therapies to decrease the frequency of VOC crisis in SCD patients.

Voxelotor + L-glutamine

Dose a (d_a_) will be represented by voxelotor 37.5 mg/kg, whilst b (d_b_) is l-glutamine 300mg/kg. Drug A is voxelotor with 17.7%, and Drug B is l-glutamine with 45% effect on VOC pain crisis reduction.

The constant potency ratio (R) for drug A= 17.7% relative to drug B= 45% is

R = $\frac{A}{B}$ = $\frac{0.177}{0.45}$ = 0.393

Loewe additivity of drug A (Dea= 17.7%) with response to drug B (Deb= 45%) is

d_a_ + d_b_D_ea_/D_eb_ = 37.5 + ($\frac{300\times 0.177}{0.45}$) = 155.5 = A

da/Dea + db/Deb = $\frac{37.5}{0.177}+\frac{300}{0.45}$ = 878.5 = B

CI = $\frac{a}{A}+\frac{b}{B}$ = $\frac{30}{155.5}+\frac{300}{878.5}$ = 0.5

The CI obtained is less than 1 and based on Table 3, CI= 0.5 is described as synergism. The combination of voxelotor and l-glutamine may increase the effectiveness of the therapies to decrease the frequency of VOC crisis in SCD patients.

L-glutamine + Crizanlizumab

Dose a (d_a_) will be represented by l-glutamine 300mg/kg and dose b (d_b_) is crizanlizumab 5.0mg/kg. Drug A is l-glutamine with a 45% effect on VOC pain crisis reduction and Drug B is crizanlizumab with 45.3%.

The constant potency ratio (R) for drug A (45%) relative to drug B (45.3%) is

R = $\frac{A}{B}$ = $\frac{0.45}{0.453}$ = 0.993

Loewe additivity of drug A (D_ea_= 45%) with response to drug B (D_eb_= 45.3%) is

da + dbDea/Deb = 300 + ($\frac{5.0\times 0.45}{0.453}$) = 304.97= A

da/Dea + db/Deb = $\frac{300}{0.45}+\frac{5}{0.453}$ = 677.70 = B

CI = $\frac{a}{A}+\frac{b}{B}$ = $\frac{300}{304.967}+\frac{5}{677.70}$ = 0.99

The CI obtained is less than 1; thus, synergism. Nevertheless, a CI of 0.99 is described as nearly additive based on Table 3. The combination of l-glutamine and crizanlizumab may cause an additive effect or improve the effectiveness of the therapies to decrease the frequency of VOC crisis in SCD patients.

Discussion

Based on the findings from our study, l-glutamine, voxelotor, and crizanlizumab were associated with both positive and negative health effects. In our discussion of the reported adverse events, the pain was segregated from the others. 

Benefits of L-Glutamine 

Our findings on the health benefits of l-glutamine therapy in SCD patients are consistent with the benefits stated in Nze et al. (2020) which include the reduction of pain crisis frequencies, hospitalization rates, and acute SCD complications. L-glutamine was also associated with reduced emergency department visits due to the decreased frequencies of uncomplicated VOCs, and the improvement of RBC levels. L-glutamine can be used as an alternate therapy for patients who are unwilling to accept hydroxyurea treatment or for patients who experienced serious adverse effects from hydroxyurea. Similarly, l-glutamine can be used as a supplementary therapy for patients with substandard responses to hydroxyurea therapy [7]. Furthermore, l-glutamine was associated with a surge in NAD and NADH [16]. Though studies suggest l-glutamine is a promising therapy for SCD, it is yet to be approved by the European Medicines Agency [16].

Benefits of Voxelotor

The benefits reported in our findings were consistent with Ali et al. (2020) and suggest that voxelotor treatment upsurges hemoglobin levels and increased oxygen capacity in the body [35]. Moreover, voxelotor was associated with other health benefits such as a reduction in (un)conjugated bilirubin level, decreased number of VOC pain crises, prevented jaundice, lessened the number of hospitalization days, normalized the reticulocyte count, relieved SCD-related stress, and enhanced the quality of life of SCD patients. In some instances, voxelotor improved the physical wellness of SCD patients by allowing them to walk a longer distance and decreasing their post-walk pause rate. Due to the effect of voxelotor on the body’s hemoglobin level and oxygen capacity, patients' need for acute or chronic blood transfusions may be minimized. This therapy may lessen the burden of transfusion reactions and iron overload [35].

Benefits of Crizanlizumab

Our findings on crizanlizumab benefits are consistent with Cisneros (2020) [16], which endorses crizanlizumab treatment. This treatment prolongs the median time between the first and second VOC pain crisis. It was also found that crizanlizumab prevents the adhesion of sickle erythrocytes to vascular endothelium. Additionally, crizanlizumab was associated with other health benefits such as reduced VOC pain episodes, hospitalization rates, and the frequency of uncomplicated VOCs.

Synergism, Antagonism, and Additivity Effects of Suggested Combination Therapies

An effect-based and dose-effect-based approach was calculated to assess the effectiveness, toxicity, and accuracy of the combined therapies to reduce VOC pain crisis among SCD patients. The CI standard unit was used to determine the synergy, additivity, and antagonistic effect of combined SCD therapies. The CI in Bliss Independence Model and the Loewe Additivity was used to determine the effect of the drugs.

Effect-based Strategy via the Bliss Independence Model

The CI measure of a combination drug effect designates a greater (CI < 1), lesser (CI > 1), or similar (CI = 1) effect compared to the expected additive effect. All three combinations, voxelotor + crizanlizumab, voxelotor + l-glutamine, and crizanlizumab + l-glutamine resulted in a CI of one which suggests these combinations would have an additive effect. This signifies the summed effect of the combined drugs would be equal to the individual effect of the therapies.

Dose-effect-based Strategy via the Loewe Additivity Model

In the Loewe Additivity Model, the CI of the suggested combined SCD therapies was calculated to determine its potential benefits. The combination therapies voxelotor + crizanlizumab and crizanlizumab + l-glutamine had a CI of 0.9 and 0.99 respectively. These results indicated a nearly additive effect and signified the combinations may result in a total effect that is equivalent to the summed effect of each drug taken independently. However, the combination of voxelotor and l-glutamine revealed a CI of 0.5, which indicated synergism. Combining voxelotor and l-glutamine may result in a greater effect than the summed individual drug effects. Based on our analysis and findings, combining the SCD therapies voxelotor and l-glutamine may be more beneficial to SCD patients. Hence, more research aimed at developing the appropriate dose for this combination therapy and the actual effect it has to reduce the VOC pain crisis should be explored. 

Best Combination Therapy

Based on our findings, voxelotor + l-glutamine had the most significant and unique health benefits reported in the studies analyzed (see Figure 5). L-glutamine and crizanlizumab had similar health benefits; however, the synergistic effect of l-glutamine + voxelotor via the Loewe Additivity model, suggests this combination may be superior. Thus, the combination between voxelotor and l-glutamine may result in increased health benefits compared to any other individual or combination therapy we studied. Further, the combined adverse events between l-glutamine and voxelotor are tolerable and the presumed toxicity levels are low (see Figure 7). The combination therapy, voxelotor + l-glutamine, is recommended to be incorporated in the treatment of SCD due to its probable increased benefit in these patients. Notably, prior to the commercial use of this combination therapy, clinical trials ought to be sought to determine the therapy’s actual effectiveness. Likewise, evaluation of the combined therapy dose level is essential to assess the toxicity and response level of the drugs being combined. The combination therapies l-glutamine + crizanlizumab and voxelotor + crizanlizumab resulted in an additive effect via the Loewe Additivity Model; thus, exclusive and comprehensive research to focus on voxelotor + l-glutamine combination therapy for the treatment of SCD is essential.

Unrelenting Pain Despite L-glutamine, Voxelotor, and Crizanlizumab Treatment

Based on our findings, pain was the most prevalent adverse event among participants receiving either l-glutamine, voxelotor, or crizanlizumab. Pain episodes were considered adverse events if they occurred during active treatment in more than ten percent of the participants. Headache, back pain, and extremity pain were reported by study participants in all three therapies. Noncardiac chest pain and (upper) abdominal pain/discomfort were reported in the voxelotor and l-glutamine studies. Arthralgia was reported in studies evaluating voxelotor and crizanlizumab. Generalized pain was reported by voxelotor participants, while chest pain and musculoskeletal pain were reported by patients receiving crizanlizumab treatment. Noteworthy, crizanlizumab treatment has been associated with pain crisis within 24 hours of infusion prior to Phase IV clinical trials and the company released a safety statement prior to marketing. In one of our studies, a patient’s initial treatment of crizanlizumab during Phase IV of the clinical trials experienced unrelenting pain. It was determined that the severe pain occurrence was consistent with Complicated Activation-Related Pseudo Allergy (CARPA), a hypersensitive reaction that is triggered or activated by the complement system. When the complement system is inappropriately activated, a natural immune defense may result in pathologic tissue damage [36].

Among the FDA-approved SCD therapies (voxelotor, crizanlizumab, and l-glutamine), crizanlizumab was associated with CARPA, triggered by the complex system activation. Although rare, there have been reports of complement system activation in SCD patients. The mechanism for its activation has not been clearly explained. Roumenina et al. (2020) reported, sC5b-9 is a marker for fatal complex system activation, that can increase steadily to 61% in SCD patients, and its activation in SCD patients is prompted by the erythrocytes. The effect of complex system activation in SCD patients is influenced by the level of dense sickle cells (DRBCs) and the reduced expression of CD59, CD55, and CD35 levels; thus, the inept regulation and upsurge in DRBCs and hemolysis. Additionally, increased levels of cS5b-9 were attributed to the activation of the complex system on endothelium cells. To note, hydroxyurea therapy has been associated with a reduction in complement system activation [36].

L-glutamine, Voxelotor, and Crizanlizumab Unlikely to Decrease Analgesic and Opioid Use

The use of analgesia and opioids to manage acute and chronic pain among adults diagnosed with SCD disease is high [37]. Moreover, there have been increased cases of overdose, abuse, and deaths related to the use of opioids reported by the United States federal and state governments. Filling opioid prescriptions at pharmacies has become more difficult and daunting due to the imposed restrictions based on abuse and overdose rates. SCD patients are being forced to search for non-opioid alternatives. Some patients who prefer to retain opioids for pain management are required to sign off on treatments and monitor usage agreements with their health care provider to obtain prescriptions [37].

Voxelotor is primarily metabolized by the CYP34A enzyme which is also the primary metabolizer of oxycodone and fentanyl. The CYP34A enzyme also plays a role in the metabolization of methadone and tramadol [38]. In the voxelotor studies we analyzed, the type and amount of opioids used were not mentioned. Thus, we could not determine if the voxelotor group attained better pain control or required fewer opioids than the placebo group. Nevertheless, the participants of all three therapies in the studies we analyzed were prescribed analgesics and or opioids during treatment to help with pain episodes. Despite a reduction in VOC pain events, SCD patients continue to experience pain crises, prompting them to depend on analgesics and opioids for pain relief. L-glutamine, voxelotor, and crizanlizumab treatment may not have a meaningful influence on the usage of analgesia and opioids in managing pain in SCD patients. Further studies are required to determine the level of impact these SCD therapies contribute to analgesic and opioid use in SCD. 

Other Adverse Events, Warnings, and Contraindications to L-glutamine, Voxelotor, and Crizanlizumab Treatment

Based on the findings in our study, all the SCD therapies evaluated were associated with adverse events apart from VOC and SCD-related pain crises. Participants being treated with l-glutamine, voxelotor, or crizanlizumab reported diarrhea, nausea, and vomiting. Both l-glutamine and crizanlizumab therapies were associated with urinary tract infections. Voxelotor and crizanlizumab were associated with upper respiratory tract infection and pyrexia. Whilst l-glutamine and voxelotor therapies were associated with fatigue and dizziness. Rash, cough, gastroenteritis, and elevated liver enzymes were reported by voxelotor participants; tachycardia, nasal congestion, and constipation by l-glutamine participants; and influenza, pneumonia, and pruritus by crizanlizumab participants.

During Phase IV clinical trials of voxelotor, one patient developed acute kidney injury, eosinophilia, and drug reaction with eosinophilia and systemic symptoms (DRESS). During Phase III clinical trials of l-glutamine, single reports of dyspepsia, hot flashes, a burning sensation in the feet, and hypersplenism were noted. During and preceding the initiation of crizanlizumab to an SCD patient during Phase IV clinical trials, the patient developed a severe drug reaction, elevated sC5b-9, persistent fever, acute chest syndrome, and post-traumatic stress with nightmares.

Other adverse events reported in the literature include renal impairment in l-glutamine therapy and in crizanlizumab, intracranial bleed, and on-site reactions such as swelling and extravasation [16]. Additionally, patients with underlying health complications, specifically organ failure, died during treatment with voxelotor, l-glutamine, and crizanlizumab. Despite the articles indicating deaths, the deaths were not associated with SCD therapies. Frequent monitoring of liver enzymes, bilirubin, alanine aminotransferase, eosinophils, and the reticulocyte count is recommended to prevent the possible development of adverse events, and negative drug reactions, and death.

L-glutamine: L-glutamine does not improve the hematological parameters because it does not alter the disease pathology in sickle red cells [24]. The use of l-glutamine is associated with side effects such as nausea, fatigue, noncardiac chest pain, and musculoskeletal pain [24]. Other adverse events associated with L-glutamine were burning sensation, constipation, dizziness, dyspepsia, hot flashes, hypersplenism, urinary tract infections, nasal congestion, tachycardia, and vomiting. L-glutamine was also linked to renal impairment, which is a common complication in SCD patients [16]. All SCD patients, taking l-glutamine should have their kidney function monitored and those with acute or chronic renal impairment should minimize or avoid l-glutamine use.

Voxelotor: Voxelotor is metabolized through oxidation and reduction reactions which are usually mediated by the CYP3A4 enzyme, with minor contributions to other enzymes such as CYP2B6, CYP2CI9, and CYP2C9. The CYP3A4 enzyme metabolizes more than 50% of all drugs; thus, the dose of voxelotor may need to be adjusted in the presents of strong CYP3A4 inhibitors or inducers. Additionally, due to the multiple metabolic pathways involved, voxelotor is vulnerable to drug-drug interactions [38].

Voxelotor is not recommended for pregnant women due to adverse effects on the fetus. For lactating mothers, breastfeeding is not recommended until two weeks from the last dose due to its association with hematopoietic system changes in the breastfed infant [25, 39]. Diarrhea is a common side effect of the voxelotor treatment [25]. Other voxelotor-related adverse health effects include skin rash, pyrexia, sore throat, swelling of the face and glands, difficulty breathing, fatigue, tarry stools, cough, fever, painful urination, increased liver enzymes, and white spots in the mouth and lips [40]. Additionally, voxelotor is associated with hypersensitivity reactions such as eosinophilia, urticaria, gastroenteritis, AKI, and upper respiratory tract infections. It can cause a hypersensitivity reaction and in one reported case, it caused DRESS which is usually attributed to anticonvulsants, sulfonamides, and allopurinol. Voxelotor is contraindicated in persons with prior drug sensitivity to voxelotor or its excipients.

Crizanlizumab: The adverse effects of Crizanlizumab include back pain, arthralgia, pyrexia, and nausea. Additionally, influenza, acute chest syndrome, respiratory failure, and aspiration are side effects of high and low dosages of crizanlizumab [41]. It is recommended that SCD patients taking crizanlizumab should receive their yearly influenza vaccine. Other notable crizanlizumab-associated adverse effects were upper respiratory infections, pruritis, persistent fever, stress, and nightmares. Crizanlizumab can also cause an infusion reaction. If the reaction is mild to moderate, temporarily stop or slow the dose rate, and if severe, discontinue immediately and consider permanent discontinuation. In addition, infusion-related reactions must be recorded as a precaution, especially in severe cases where hospitalization is imminent [41]. The association between crizanlizumab and pain events within 24 hours of administration is not adequately known, hence, in-depth research is required. Patients undergoing crizanlizumab treatment should also be monitored for infusion site reactions such as extravasation, pain, swelling, and other infusion-related reactions [14].

Conversely, the manufacturer’s label stated crizanlizumab has no contraindications, however, cautions against its use with corticosteroids and warns it may cause fetal harm and immunogenicity. The possibility of immunogenicity is dependent on the specificity and sensitivity of the biochemical and protein nature of crizanlizumab [41]. The occurrence of antibodies may also be influenced by other factors such as metallurgy methodology, timing and collection of specimen collection, concomitant medications, and underlying health issues [41]. Crizanlizumab causes platelets to clump in EDTA tubes, invalidating platelet values; as a result, citrate tubes are recommended, or tests should be processed as soon as possible to alleviate this potential problem.

During treatment, frequent monitoring and follow-up sessions are required to be able to detect the presence of antibodies against the crizanlizumab treatment and neutralize them to enable the safe and long-term administration of crizanlizumab therapy [13]. Crizanlizumab is not advised for pregnant women because of potential harm to the fetus, however, in severe cases, patients may use crizanlizumab [42]. It is also associated with intracranial bleed since it interferes with the functioning of the platelets through its effect on selectins [16]. SCD patients with a history of intracranial bleed, taking blood thinners or anticoagulation agents should not be prescribed crizanlizumab.

Limitations

All of the RCTs we analyzed employed different inclusion criteria despite using the same patient population (SCD patients with two or more VOC in the previous year). This limited the generalizability of the results to a broader population and comparison of the results to other similar studies. A great proportion of SCD participants in all of the RCTs were taking hydroxyurea simultaneously. Although the writers stated there was no significant difference between SCD participants taking hydroxyurea and those who were not, further studies should limit this potential confounding factor. It’s important also to analyze whether hydroxyurea causes a synergistic, antagonistic, or additive effect when combined with other approved SCD therapies. The small sample sizes of the RCTs we analyzed limit the generalizability of the findings. Study seven was an uncontrolled case series, hence, the data cannot be compared with data obtained from RCTs and the results should not be generalized [29]. Underlying health issues in some of the participants led to the delay or discontinuation of treatment. In some cases, the patients with severe underlying health issues such as organ failure succumbed during active treatment.

## Conclusions

With the life expectancy of persons with SCD in the United States continuing to be significantly below the national average, greater emphasis should be taken to develop cures and medications to alleviate, if not, minimize morbidity and early mortality in SCD patients. Since one of the leading causes of death in SCD is VOC acute and chronic effects, the drugs l-glutamine and crizanlizumab have been shown to decrease the occurrences of VOC episodes. Alternatively, voxelotor improves hemoglobin concentrations. Though these medications are less likely to curb the use of analgesics and opioids to manage pain in persons with SCD, each improves their general health. However, based on the dose-effect-based strategy via the Loewe Additivity model, SCD patients may achieve greater benefit from an l-glutamine plus voxelotor combination therapy than monotherapy of l-glutamine, voxelotor, or crizanlizumab. Therefore, future studies analyzing drug combinations of FDA-approved therapies should focus on l-glutamine plus voxelotor. New and improved treatment for SCD patients is a necessity long overdue.
